# Characterization of the genomic sequence of an iflavirus, a protoambidensovirus, and of three microviruses detected in mosquitoes (*Aedes albopictus* and *Culex quinquefasciatus*)

**DOI:** 10.1128/mra.00263-25

**Published:** 2025-06-20

**Authors:** Sarah François, Aymeric Antoine-Lorquin, Doriane Mutuel, Patrick Makoundou, Marco Perriat-Sanguinet, Sandra Unal, Hélène Sobry, Anne-Sophie Gosselin-Grenet, Mylène Ogliastro, Mathieu Sicard, Mylène Weill, Célestine Atyame, Christophe Boëte

**Affiliations:** 1 DGIMI, Univ Montpellier, INRAE 27037, Montpellier, France; 2 Department of Biology, University of Oxford 98459, Oxford, United Kingdom; 3 ISEM, Univ Montpellier, CNRS, IRD, EPHE 27037, Montpellier, France; 4 UMR PIMIT (Processus Infectieux en Milieu Insulaire Tropical) CNRS 9192, INSERM 1187, IRD 249, Université de La Réunion 57493, Saint-Denis, France; Katholieke Universiteit Leuven, Leuven, Belgium

**Keywords:** virus, mosquito, *Aedes*, *Culex*, iflavirus, microvirus, protoambidensovirus

## Abstract

We report the complete CDS of five viruses: XiangYun picorna-like virus 4 (*Iflaviridae*), *Protoambidensovirus dipteran1* (*Parvoviridae*), and three microviruses (*Microviridae*), detected by viromics surveillance of *Aedes albopictus* and *Culex quinquefasciatus* from Réunion Island. The protoambidensovirus detected in *A. albopictus* belongs to a clade previously reported only in *Culex pipiens*.

## ANNOUNCEMENT

Our knowledge of mosquito viruses remains limited, although they can modulate the transmission of arboviruses (1). We processed 13 *Aedes albopictus* and 9 *Culex quinquefasciatus* larvae pools from 14 different sites across Réunion Island in 2019. Morphological characteristics were used for species identification, and larvae were pooled by location. Samples weighted between 0.5 g and 1.49 g and stored at −80°C. Viromes were obtained as described in Reference 2. We followed manufacturers’ instructions and default parameters except where otherwise noted. Briefly, samples were homogenized in Hanks' Balanced Salt Solution (HBSS) by a bead-beater, filtered through a 0.45 µm filter, and centrifuged at 140,000 g for 2.5 h. Pellets were resuspended and digested by DNase I and RNase A incubation at 37°C for 1.5 h. RNA and DNA were extracted using the NucleoSpin kit. Reverse transcription was performed by SuperScript III, cDNAs were purified by QIAquick PCR Purification Kit, and dsDNA was synthesized by Klenow DNA polymerase I. DNA was amplified by random PCR (2), and PCR products were purified using NucleoSpin gel and a PCR clean-up kit. Libraries were prepared using NEBNext Ultra DNA PCR free with Illumina adapter kit without fragmentation step and sequenced on Illumina HiSeq3000 in 2 × 150 bp paired-end mode.

Adaptors were removed, and reads were filtered for quality (q30 and read length >45 nt) using cutadapt 2.19 (3). A total of 10,150,317 reads (min: 198,733, average: 461,378, and max: 827,929 reads per pool) were assembled into contigs by MEGAHIT 1.2.9 (4). Taxonomic assignment was achieved using DIAMOND 0.9.30 against the nr protein database (5). Genome coverage was assessed by mapping cleaned reads from each sample against contigs using Bowtie2 3.5.1 (end-to-end very sensitive) (6). Open reading frames (ORFs) were identified using ORF finder (cutoff >400 nt) on Geneious Prime 2024.0.5 (7) and were annotated by blastn query-centered alignment against RefSeq viral database (October 2024). Genome circularization was performed using Simple-Circularise (overlap >20 nt).

We reconstructed the complete circular genomes of three bacteriophages from the *Microviridae* family (Gokushovirinae subfamily). They share 54.5%–75.0% of amino acid identity in the major capsid and replication-associated proteins with their closest relatives isolated from water environments (Table 1). There are currently no genome similarity species demarcation criteria defined by the ICTV for the *Microviridae* family (8). They may infect *Aedes* microbiota, although their presence could be due to environmental or diet contamination.

**TABLE 1:** Information on the five viruses reconstructed from mosquito viromic data

Virus	GenBank accession number	Genome		Coverage			Putative proteins			Closest identified relatives			
		Size (nt)	%GC	Samples from which viral genomes were reconstructed	Average	Number of reads	Name	Size (nt)	Size (aa)	Virus name	Accession number	Pairwise identity (aa)	Source
*C. quinquefasciatus* associated densovirus, isolate 2019_VP12-D87	PQ041300	5,459	36.7	Cxqfts virome sample 3 (D87) SRR29133503	12,521	5,01,573	NS1	687	229	*Culex pipiens* densovirus	FJ810126	94.7	*Culex pipiens*
							NS2	372	214			92.2	
							NS3	642	124			42.4	
							VP	2,208	736			90.5	
*C. quinquefasciatus* associated iflavirus, isolate 2019_VP12-D85	PQ041301	10,074	36.9	Cxqfts virome sample 1 (D85) SRR29133486	2,777	1,94,020	Polyprotein	9,231	3,077	XiangYun picorna-like virus 4	OL700176	98.5	*Culex theileri*
*A. albopictus* associated microvirus 1, isolate 2019_VP12-D83	PQ041302	4,365	43.9	Aedes virome sample 11 (D83) SRR29133492	243	8,198	Major capsid protein	1,650	550	*Microvirus sp.*	MT309944	75.0	Wastewater
							DNA pilot protein	678	226	*Microviridae sp.*	OR998997	43.3	Bacteria
							Internal scaffolding protein	429	143	*Microvirus sp.*	MT309947	54.2	Wastewater
							Replication initiator protein	1,023	341	Arizlama microvirus	MW697615	54.9	Freshwater
*A. albopictus* associated microvirus 2, isolate 2019_VP12-D75	PQ041303	4,387	46.3	Aedes virome sample 3 (D75) SRR29133501	62	2,200	Major capsid protein	1,635	545	*Microviridae sp.*	MH572427	67.3	*Ciona robusta*
							DNA pilot protein	801	267	*Microvirus sp.*	OR349779	23.4	Mollusc
							Internal scaffolding protein	474	158	*Microviridae sp.*	MH616902	65.8	Fish
							Replication initiator protein	747	249	*Microvirus sp.*	MT309954	74.7	Wastewater
*A. albopictus* associated microvirus 3, isolate 2019_VP12-D82	PQ041304	4,813	50.5	Aedes virome sample 10 (D82) SRR29133485	197	6,618	Major capsid protein	1,653	551	*Microviridae sp.*	MH617066	73.4	Fish
							DNA pilot protein	795	265	*Microviridae sp.*	MH617641	37.6	Fish
							Internal scaffolding protein	444	148	*Microviridae sp.*	MH617066	61.9	Fish
							Replication initiator protein	783	261	*Microvirus sp.*	MT310309	68.6	Wastewater

We report a complete CDS of XiangYun picorna-like virus 4 (*Iflaviridae* and *Iflavirus*; Table 1). It shares a polyprotein pairwise identity of 98.5% with its closest relative reported in *Culex theileri* from China (9). This lineage clustered with viral taxa isolated from diptera (mosquitoes and true flies; Supplementary Figure [SF] 10.6084/m9.figshare.28565888), indicating that it is likely specific to dipterans. Further work is needed to determine the prevalence and impact of this virus in mosquitoes.

**Fig 1:**
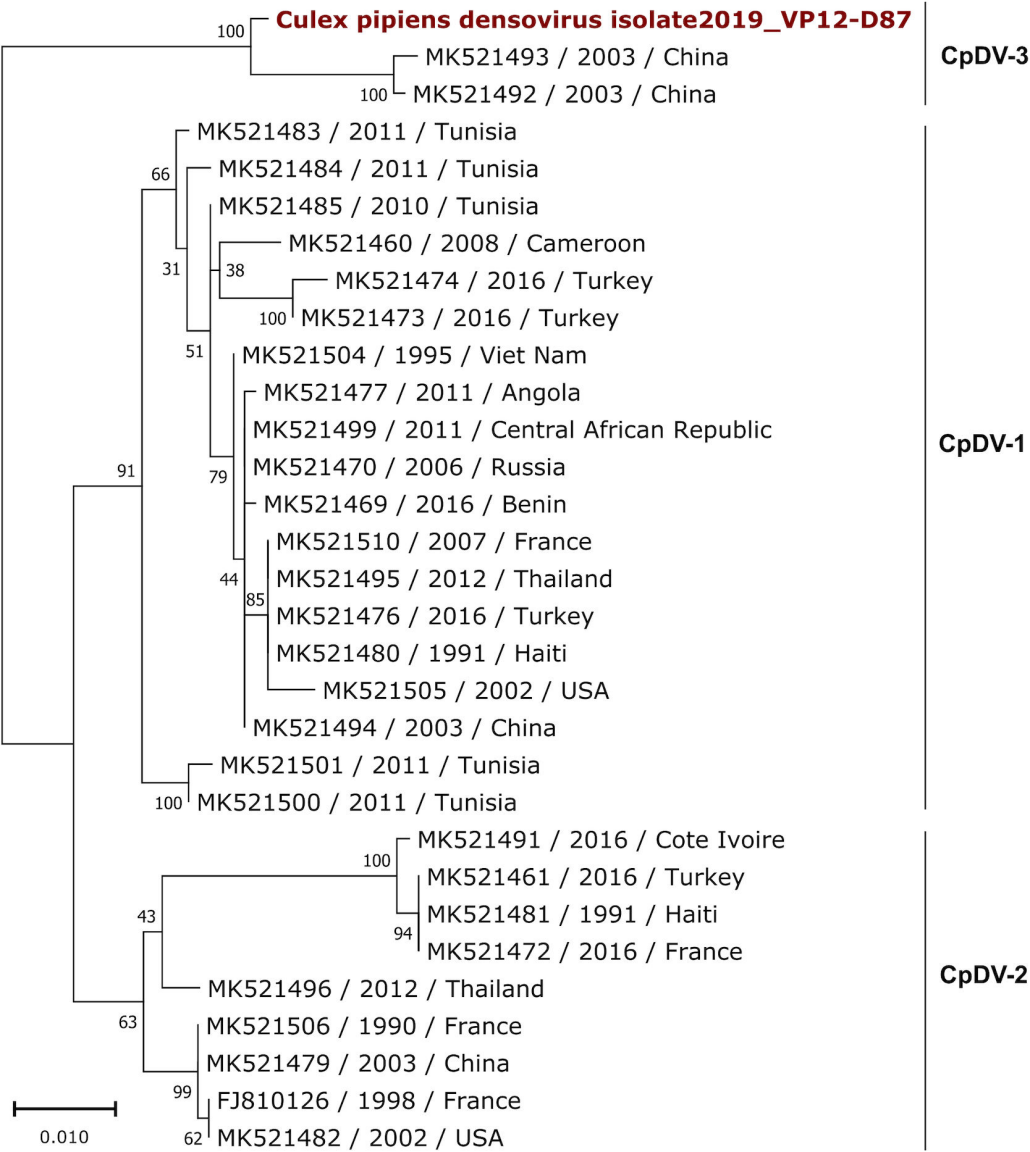
Maximum likelihood phylogenetic tree based on the NS1 nucleotide sequences of 31 *Protoambidensovirus dipteran* 1 (common virus name: *Culex pipiens* densovirus [CpDV]) sequences. The alignment of 892 nucleotides in length was produced by MAFFT v7.450 using the G-INS-i algorithm. The GTR + G + I nucleotide substitution model was selected using MEGAX 10.2.6 (13), and the tree was reconstructed by RAxML 8.2.11 (14). The tree is mid-point rooted and visualized using MEGAX. Bootstrap values (1,000 replicates) are indicated at each node. The scale bar corresponds to nucleotide substitutions per site. The CpDV sequence obtained from our sample is colored in red.

We report a complete CDS of the mosquito infecting *Protoambidensovirus dipteran* 1 species (*Parvoviridae* and *Protoambidensovirus*; Fig. 1). It shares 89.0% genome-wide nucleotide identity with its closest relative (Table 1) (10). It belongs to the CpDV-3 clade previously only represented by sequences from China (11). The reported CpDV host range only includes *Culex pipiens* (11, 12); its broadening to *A. albopictus* warrants further confirmation.

## Data Availability

The genomic sequences of the five full-length viral genomes or CDS have been deposited at GenBank under the accession numbers PQ041300 to PQ041304. High-throughput sequencing reads were deposited in SRA under the accession no. SRR29133481 to SRR29133504 under PRJNA1114772 BioProject.
